# Diffusion of Copper Ions in the Lattice of Substituted Hydroxyapatite during Heat Treatment

**DOI:** 10.3390/ma15165759

**Published:** 2022-08-20

**Authors:** Natalia V. Bulina, Natalya V. Eremina, Olga B. Vinokurova, Arcady V. Ishchenko, Marina V. Chaikina

**Affiliations:** 1 Institute of Solid State Chemistry and Mechanochemistry, Siberian Branch of the Russian Academy of Sciences, Kutateladze Str. 18, 630128 Novosibirsk, Russia; 2 G.K. Boreskov Institute of Catalysis, Siberian Branch of Russian Academy of Sciences, Pr. Akad. Lavrentieva 5, 630090 Novosibirsk, Russia

**Keywords:** mechanochemical synthesis, hydroxyapatite, copper, copper oxide, substitution, heat treatment

## Abstract

The doping of hydroxyapatite with various substituent ions can give this material new and useful properties. Nonetheless, local distortions of structure after doping can change the properties of the material. In this work, the thermal stability of copper-substituted hydroxyapatite synthesized by the mechanochemical method was investigated. In situ diffraction analyses showed that copper ion diffusion during the heating of Cu-substituted hydroxyapatite promotes phase transformations in the substituted hydroxyapatite. The behavior of copper ions was studied in samples with ratios (Ca + Cu)/P = 1.75 and 1.67. It was found that in both cases, single-phase Cu-substituted hydroxyapatite with the general formula Ca_10−x_Cu_x_(PO_4_)_6−y_(CO_3_)_y_(OH)_2−y_O_y_ is formed by the mechanochemical synthesis. When heated at approximately 600–700 °C, the lattice loses copper cations, but at higher temperatures, CuO diffusion into the hydroxyl channel takes place. Cuprate-substituted hydroxyapatite with the general formula Ca_10_(PO_4_)_6_(OH)_2−2x_(CuO_2_)_x_ forms in this context. At 1200 °C, the sample is single-phase at (Ca + Cu)/P = 1.75. Nonetheless, slow cooling of the material leads to the emergence of a CuO phase, as in the case of (Ca + Cu)/P = 1.67, where the material contains not only CuO but also Cu-substituted tricalcium phosphate. In the manufacture of ceramic products from Cu-substituted hydroxyapatite, these structural transformations must be taken into account, as they alter not only thermal but also biological properties of such materials.

## 1. Introduction

Hydroxyapatite (HA), i.e., Ca_10_(PO_4_)_6_(OH)_2_, is widely used in various fields of medicine: in traumatology, orthopedics, craniofacial surgery, dental technologies, medical treatments, and cosmetology, as a means of targeted drug delivery. HA is a suitable material for the development of biocompatible ceramic products, composites, bone defect fillers, medical cements, and implant coatings [1,2,3].

**Figure 1:**
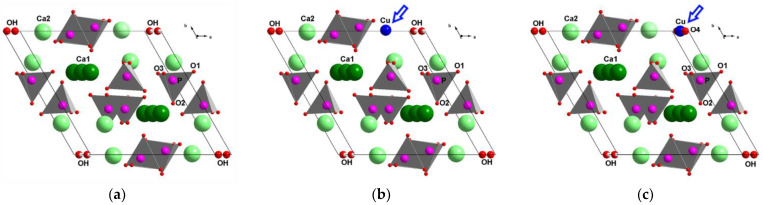
Crystal structure of stoichiometric HA (**a**) and of Cu-HA, featuring the localization of the copper cation either at the site of the calcium cation (**b**) or in the hydroxyl channel (**c**). The arrow indicates the position of the copper atom.

HA (Figure 1a) crystalizes in hexagonal syngony with space group *P*6_3_/*m* [4]. The HA unit cell contains 10 calcium cations located at two nonequivalent positions: four cations at the Ca1 site and six cations at the Ca2 site, which are surrounded by nine and seven oxygen atoms, respectively. In addition to calcium ions, the HA unit cell contains six phosphate and two hydroxyl groups. The latter are located on the *c* axis in a hexagonal channel formed by calcium ions and by oxygen ions from phosphate tetrahedrons. The crystal lattice of HA is unique in that it offers ample opportunities for substitutions and for the formation of solid solutions. Some or even all ions can be substituted, and replacement with isovalent or heterovalent ions of other chemical elements or their chemical groups is possible [5]. Doping of HA with copper ions gives an antibacterial property to HA-based materials, which reduces the risk of inflammation after implantation [6]. In addition, copper cations induce protein absorption, osteogenic differentiation, and bone-like apatite nucleation and growth at an implant site [7]. These effects stimulate the growth of new bone tissue and accelerate the healing process, both of which are important for surgical applications.

There exists a study indicating that in samples of Cu-substituted HA (Cu-HA) obtained by the precipitation technique, after calcination at 400 °C, copper ions can replace calcium ions (Figure 1b) up to substitution degree x = 1.5 with the formation of Ca_10−x_Cu_x_(PO_4_)_6_(OH)_2_ [8]. Solid-phase mechanochemical synthesis in a planetary ball mill allows the obtaining of Cu-HA with substitution degree x = 2 [9]. Heat treatment at >1000 °C makes it possible to stabilize copper ions in the hydroxyl channel in the form of linear oxocuprate groups (Figure 1c) with the formation of the Ca_10_(PO_4_)_6_(OH)_2−2x_(CuO_2_)_x_ structure, where x ≤ 0.6 [10,11,12,13,14,15,16]. Thus, the following mechanisms of substitution in HA are possible for the copper cation:

Ca^2+^ → Cu^2+^ (1)

2OH^−^ → (CuO_2_)^2−^ (2)

The main areas of application of apatite materials necessitate a heat treatment (usually high-temperature) procedure during the manufacture of a product. The authors of ref. [8] report that after heat treatment at 700 °C, orthophosphates Ca_3_Cu_3_(PO_4_)_4_ and Ca_19_Cu_2_(PO_4_)_14_ form in Cu-HA with a high degree of substitution. The absence of high-temperature treatment is necessary for the practical application of such a material because a change in the phase composition of the material affects its properties. Therefore, it is necessary to know the limitations of the heat treatment of Cu-HA.

The aim of this work is to investigate the evolution of phase composition during heat treatment of Cu-HA samples prepared by the mechanochemical method with a low degree of substitution. In situ high-temperature experiments on these samples were carried out for the first time in the present study.

## 2. Materials and Methods

**Figure 2:**
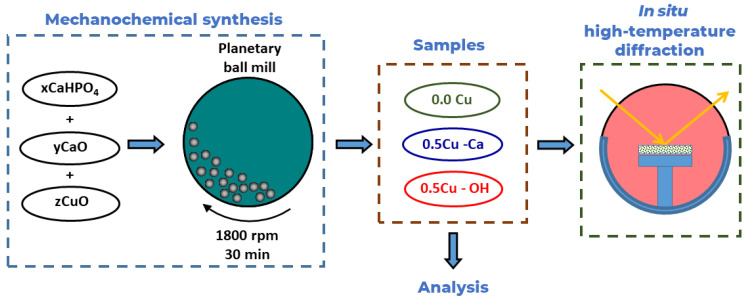
Schematic representation of the experiments.

Schematic representation of the experiments is shown in Figure 2. At the first stage, mechanochemical synthesis of three samples and their analyses were carried out. Then, in situ high-temperature experiments were conducted on the synthesized samples.

The following reagents were used as starting components for the mechanochemical synthesis: anhydrous calcium hydrogen orthophosphate CaHPO_4_, annealed calcium oxide CaO, and copper oxide CuO. All reagents were of chemically pure grade and were produced by Vekton (St. Petersburg, Russia). The ratio of components for the synthesis was chosen in accordance with reactions 1–3 given in Table 1. 

To obtain substituted-HA samples, the amount of the dopant (CuO) was selected under the assumption that copper ions replace either calcium ions (sample 0.5Cu-Ca) or hydroxyl groups (sample 0.5Cu-OH). The mechanochemical synthesis of the samples was carried out in an AGO-2 planetary ball mill [17] in two water-cooled steel drums (volume: 150 mL), with steel balls weighing 200 g, at a drum rotation speed of 1800 rpm in ambient air. The weight ratio of the reaction mixture to the balls was 1:20, and the processing time of the initial mixture in the mill was 30 min. Before the synthesis, the working zone of the mill was lined with a reaction mixture of the same composition.

X-ray powder diffraction (XRPD) patterns of the obtained samples were recorded on a D8 Advance powder diffractometer (Bruker, Germany) with Bragg–Brentano geometry using CuKα radiation. In situ high-temperature experiments were conducted in an HTK 1200N chamber (Anton Paar, Graz, Austria) with a corundum carrier in the air atmosphere. Heating was carried out stepwise at a heating rate of 0.5 °C/s with exposure to a given temperature for 600 s. The diffraction patterns were detected with a step of 0.02° and a scan speed of 1 s per step. X-ray phase analysis of the compounds was performed using the ICDD PDF-4 database of XRPD patterns (a 2011 release). Refinement of unit cell parameters and of crystallite size, calculations of phase concentrations, and refinement of structural parameters (atomic coordinates and occupancies) were carried out by the Rietveld method in the Topas 4.2 software (Bruker, Germany). The instrumental contribution was modeled by the fundamental parameter method. Initial structural data on the Cu-HA phase were borrowed from ref. [11].

Transmission electron microscopy (TEM) and high-resolution TEM (HRTEM) images were obtained using a Themis-Z 3.1 microscope (TFS, Waltham, MA, USA) at an accelerating voltage of 200 kV. The microscope is equipped with a field emission cathode having a monochromator and with two aberration correctors. Energy-dispersive X-ray microanalysis of elemental composition of the samples was performed on a four-segment Super-X detector (with an energy resolution of ~120 eV) in scanning dark-field mode with the construction of maps of distributions of elements by means of characteristic lines of the spectrum from each point in an analyzed region. Samples for the analysis were dispersed by ultrasonication and deposited from alcohol on perforated aluminum grids covered with a thin carbon mesh.

Fourier transform infrared (FTIR) spectra were acquired with the help of an Infralum-801 spectrometer (Simex, Russia). The samples were prepared by the KBr pellet method.

## 3. Results and Discussion

### 3.1. Mechanochemical Synthesis

**Figure 3:**
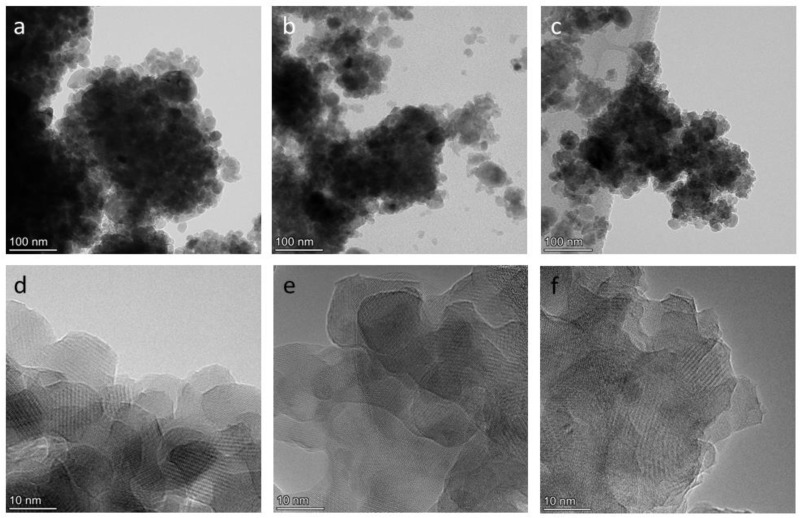
TEM (**a**–**c**) and HRTEM (**d**–**f**) images of synthesized samples 0.0Cu (**a**,**d**), 0.5Cu-Ca (**b**,**e**), and 0.5Cu-OH (**c**,**f**).

**Figure 4:**
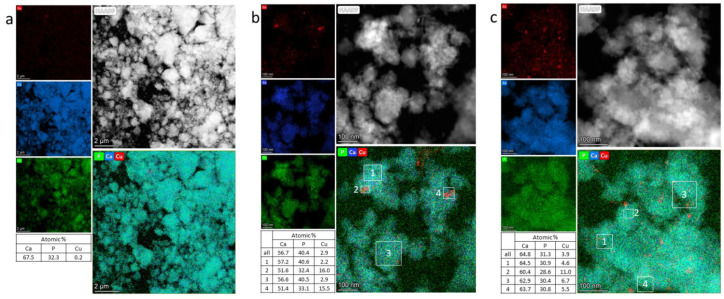
Distribution maps of elements across particles in synthesized samples 0.0Cu (**a**), 0.5Cu-Ca (**b**), and 0.5Cu-OH (**c**).

Figure 3 and Figure 4 depict electron micrographs of the synthesized samples and distributions of elements across particles in the synthesized samples. All the samples proved to be aggregates of nanoparticles ranging in size from 50 to 1000 nm. The nanoparticles are crystalline, with a size of 20–100 nm. X-ray microanalysis of Cu-doped samples showed that calcium and phosphorus are distributed evenly, but copper behaves differently. Most of the aggregates in samples 0.5Cu-Ca and 0.5Cu-OH contain 3 at.% and 4 at.% of evenly distributed copper, respectively. At the same time, nanoparticles with a high copper content are present (Figure 4b,c). The average Ca/P ratios in samples 0.5Cu-Ca and 0.5Cu-OH are 1.4 and 2.1, respectively, whereas the average (Ca + Cu)/P ratio is 1.5 and 2.3. These values differ from theoretical ones (see Table 1), possibly because this method of analysis is local; micron-sized particles are not analyzed in this case. In sample 0.0Cu, where no copper was used in the synthesis, 0.2 at.% of copper was detected (Figure 4a), which is a background value and is explained by the presence of copper in components of the optical part of the microscope.

**Figure 5:**
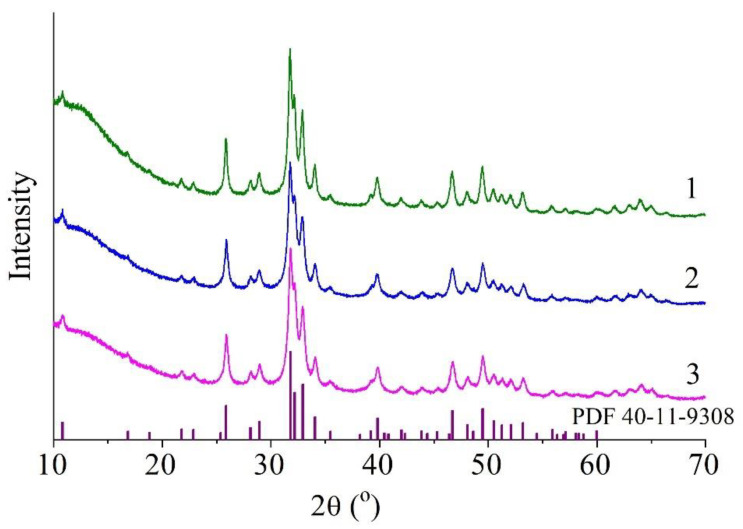
XRPD patterns of samples 0.0Cu (**1**), 0.5Cu-Ca (**2**), and 0.5Cu-OH (**3**) after synthesis according to reactions (**1**–**3**) (Table 1), as detected at room temperature.

Figure 5 shows diffraction patterns of the samples after the mechanochemical synthesis. In the diffraction patterns, there are reflections characteristic of the HA phase, corresponding to card PDF 40-11-9308, indicating that the synthesized substances are single-phase. The absence of any reflections of impurity phases confirmed that the expected substances were obtained in accordance with the above reactions (Table 1). From Table 2, it can be concluded that the lattice parameters of HA in the Cu-HA samples undergo the dynamics similar to those of unsubstituted-HA parameters. In both substituted-HA samples, parameters *a* and *c* are slightly less than those of unsubstituted HA. Such a situation can be observed when calcium cations are substituted [8,9] and is explained by the smaller ion radius of the dopant: r_Ca2+_ = 0.100 nm versus r_Cu2+_ = 0.073 nm. From Table 2, it also follows that the introduction of the dopant reduces the crystallinity of the material in question. In the lattice, the substituent ions represent point defects that complicate crystallite growth.

**Figure 6:**
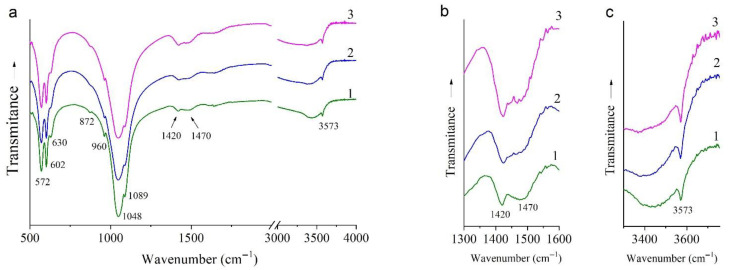
FTIR spectra of samples 0.0Cu (**1**), 0.5Cu-Ca (**2**), and 0.5Cu-OH (**3**). (**a**) General view; (**b**) enlarged view of the 1300–1600 cm^−1^ region; (**c**) enlarged view of the 3300–3750 cm^−1^ region.

FTIR spectra of the synthesized samples are identical (Figure 6). They contain an absorption pattern corresponding to HA structure: absorption bands of the phosphate ion (572, 602, 960, 1048, and 1089 cm^−1^) and of the hydroxyl group (630 and 3573 cm^−1^). The same intensity of the hydroxyl group’s absorption bands among all the synthesized samples (Figure 6c) indicates hydroxyl’s equal concentrations; therefore, substitution of the hydroxyl group did not occur in the 0.5Cu-OH sample, where a twofold decrease in the concentration of OH groups was expected according to the reaction (Table 1, reaction 3). Hence, for the 0.5Cu-Ca sample, the reaction proceeded as expected (Table 1, reaction 2). By contrast, the synthesis of the 0.5Cu-OH sample, where hydroxyl group substitution was assumed (Table 1), did not match the desired reaction (Table 1, reaction 3). To understand what happened in the 0.5Cu-OH sample, we created the following line of reasoning. 

In accordance with the literature data given above, the copper cation introduced during the synthesis can occupy the position of either the hydroxyl group or the phosphate group (Figure 1b,c). If the former scenario does not materialize in the 0.5Cu-OH sample, then the latter one does. When copper ions are located at the calcium site, coefficient k for this sample can be calculated as follows: k = (Ca + Cu)/P = 1.75, which exceeds k necessary for the formation of HA structure (1.67); therefore, such apatite cannot form. Nonetheless, k of 1.67 can be obtained by increasing the concentration of anions by means of carbonate groups, so that k = (Ca + Cu)/(P + C) = 1.67. This idea is supported by knowledge about the process of HA mechanochemical synthesis [18]. If the synthesis is carried out in ambient air with calcium oxide as the calcium source, then the following reactions take place on the particle surface:

CaO + H_2_O = Ca(OH)_2_ (3)

Ca(OH)_2_ + CO_2_ = CaCO_3_ + H_2_O (4)

The second reagent used—the phosphate source CaHPO_4_—reacts with calcium carbonate, and as a result, carbonate ions have an opportunity to become integrated into the HA crystal lattice:

(6 − x)CaHPO_4_ + (4 − x)CaO + xCaCO_3_ = Ca_10−__x_(PO_4_)_6−__x_(CO_3_)_x_(OH)_2−__x_ + 2H_2_O (5)

where x ≤ 1.

Since air access during the synthesis is limited (the synthesis is performed in closed drums), only a small amount of carbonate gets incorporated [19,20]. These anions occupy the position of phosphate groups, as indicated by FTIR spectroscopy data (Figure 6b). In the spectra of samples 0.0Cu and 0.5Cu-Ca, low-intensity absorption bands at 872, 1420, and 1470 cm^−1^ are seen. According to ref. [21], these bands belong to the carbonate ion at the position of a phosphate group. In the case of the 0.5Cu-OH sample, absorption bands of the carbonate ion are more intense (Figure 6b); hence, there is another factor stimulating the carbonate incorporation. The reason is the necessity to increase the amount of anions to reach the same value as HA has: k = (Ca + Cu)/(P + C) = 1.67. In this regard, the general chemical formula for the 0.5Cu-OH sample should be Ca_10−x_Cu_x_(PO_4_)_6−y_(CO_3_)_y_(OH)_2−y_O_y_, where y = x/1.67.

From the above findings, we can conclude that after 30 min of the mechanochemical processing of the mixtures according to reactions 2 and 3 (see Table 1), in both cases, the type of Cu-HA forms, where copper ions substitute for calcium ions. Under the tested conditions of mechanochemical synthesis, the replacement of hydroxyl groups with copper cations was unsuccessful. A possible reason is that a vacancy must be present to accommodate the oxocuprate group in the hydroxyl channel; this means that an OH group must be removed, which is possible only at high temperatures [22,23]. Under our conditions of mechanochemical synthesis (power of the mill, speed of drum rotation, and cooling conditions), an insufficient amount of energy is released via the collision and friction of the balls. A local increase in temperature can reach 600 °C [24]; however, effective dehydroxylation in ambient air requires a temperature of at least 1000 °C [22], consistent with data from refs. [10,11,12,13,14,15,16], where a temperature of 1100 °C was ensured for the synthesis of Cu-HA containing oxocuprate groups in the hexagonal channel (Figure 5 and Table 2).

### 3.2. In Situ Diffraction

#### 3.2.1. The 0.0Cu Sample

**Figure 7:**
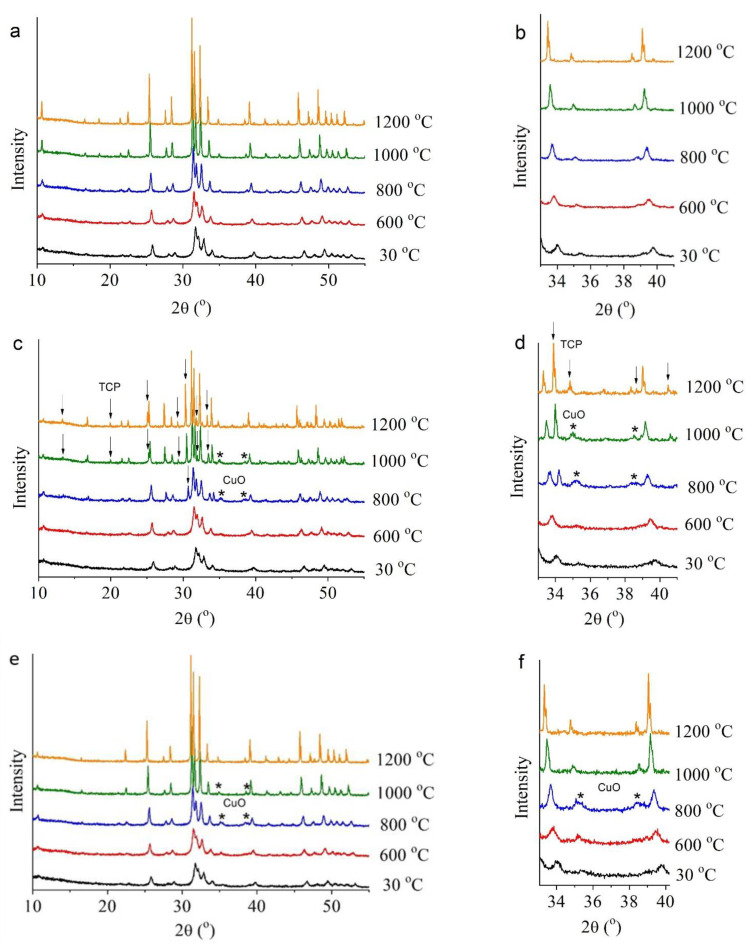
XRPD patterns recorded during in situ heating of samples 0.0Cu (**a**,**b**), 0.5Cu-Ca (**c**,**d**), and 0.5Cu-OH (**e**,**f**) in ambient air. ↓—TCP reflections; *—CuO reflections.

Examination of the synthesized samples by high-temperature in situ XRPD analysis revealed that they behave differently when heated to 1200 °C. According to the in situ XRPD data, the unsubstituted-HA sample (0.0Cu) is stable over the entire analyzed temperature range (Figure 7a). With an increase in temperature, rising intensity of reflections was observed, as was a decrease in their half-width, indicating the growth of crystallites during the heating of the sample. Reflections of any additional phases were not detectable. Due to thermal expansion, the lattice parameters of the unsubstituted-HA sample enlarged with the increasing temperature (Figure 7a); parameter *c* deviated from a linear dependence in the temperature range 1000–1200 °C. The process stopped being linear because in this temperature range, HA begins to rapidly lose hydroxyl groups located on the *c* axis [22]; these groups leave vacancies and O^2−^ behind in accordance with the equation:

Ca_10_(PO_4_)_6_(OH)_2_ → Ca_10_(PO_4_)_6_(OH)_2−2x_O_x_□_x_ + xH_2_O (6)

where □ is a vacancy. In this context, two hydroxyl groups give rise to one water molecule:

2OH^−^ → H_2_O + O^2−^ (7)

#### 3.2.2. The 0.5Cu-Ca Sample

As for the 0.5Cu-Ca sample, where the dopant was introduced under the assumption of substitution of calcium ions [k = (Ca + Cu)/P = 1.67], this material is less thermally stable. At 700 °C, a CuO release was observed, whose concentration increased at 800 °C and then began to diminish with the increasing temperature up to 1000 °C (Figure 7, Table 3). At higher temperatures, no CuO reflections were detectable in the diffraction pattern. 

In addition, starting from 800 °C, a large amount of another impurity phase emerged—β-Ca_3_(PO_4_)_2_—the concentration of which rose with the increasing temperature and reached a maximum at 1200 °C, namely 33.7 wt% (Table 3). Evidently, the drop of the CuO concentration at high temperatures down to complete disappearance points to the diffusion of copper ions into the lattice of the observed calcium phosphates [HA and β-Ca_3_(PO_4_)_2_]. It is known that substitution of some cations with copper is possible, not only in HA but also in Ca_3_(PO_4_)_2_ [8,25].

**Figure 8:**
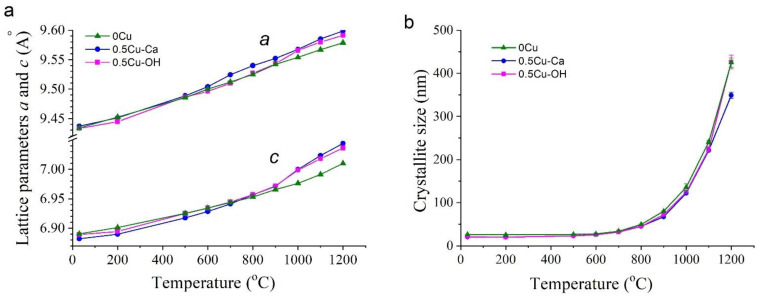
Shifts in lattice parameters *a* and *c* (**a**) and in the coherent scattering region (**b**) of the HA phase in the synthesized samples during heat treatment in the air atmosphere.

Judging by the obtained data, the copper ions introduced into the sample under the assumption of substitution of calcium ions (see Table 1) indeed occupied the positions of calcium ions after the synthesis, thereby forming the structure of substituted HA. This crystal lattice is stable up to 600 °C. At 700 °C, copper ions begin to depart from the HA lattice, thus leaving vacancies behind (former positions of calcium ions) and forming the CuO phase, possibly by combining with anion O^2−^ from the hydroxyl channel. In this scenario, the process should be accompanied by dehydration and additional formation of vacancies in the hydroxyl channel. When copper cations leave the Cu-HA lattice, the k value, (Ca + Cu)/P, becomes less than 1.67. A slight increase in lattice parameter *a* of the 0.5Cu-Ca sample, as compared with the behavior of this parameter in unsubstituted HA, is evidently attributable to the presence of vacancies in the HA lattice (Figure 8a). The highest concentration of CuO in the 0.5Cu-Ca sample is observed at 800 °C. Therefore, the maximum number of vacancies should also be seen at this temperature. The high concentration of vacancies stimulates structural transformation; thus, at 800 °C, the β-Ca_3_(PO_4_)_2_ phase appears, which has k = 1.5. This observation is consistent with the results of Destainville et al. [26], who have demonstrated that HA with k = 1.5 transforms into β-Ca_3_(PO_4_)_2_ at 750 °C. In our experiment, a complete transition of HA to β-Ca_3_(PO_4_)_2_ was not observed. The formation of 17 wt% β-Ca_3_(PO_4_)_2_ was detected at 800 °C because k is 1.58 for 0.5Cu-Ca.

Starting at 900 °C, the concentration of released CuO begins to diminish until it disappears at 1100 °C. This observation indicates that copper ions return to the HA lattice and probably to β-Ca_3_(PO_4_)_2_. It is known that prolonged 1100 °C annealing of a mixture of reagents containing CuO causes the formation of HA having linear oxocuprate groups in the hydroxyl channel [12,13]; this arrangement enlarges the lattice parameters of HA [12]. In our case, the lattice parameters of the 0.5Cu-Ca sample also increase in comparison with unsubstituted HA (Figure 8a). Accordingly, it can be assumed that copper ions of the 0.5Cu-Ca sample at 900–1000 °C are localized to the hydroxyl channel owing to the presence of vacancies of hydroxyl groups, thereby forming the Ca_10_(PO_4_)_6_(OH)_2−2x_Cu_x_O_2x_ structure. The substitution mechanism will be as follows:

□_OH_ + O^2−^ + CuO → (O–Cu–O)^2−^ (8)

#### 3.2.3. The 0.5Cu-OH Sample

In the in situ XRPD experiment on the 0.5Cu-OH sample, where (Ca + Cu)/P = 1.75, CuO separated out as well, which began at a lower temperature: starting from 600 °C (Figure 7, Table 3). Perhaps the reason is the presence of a higher concentration of the carbonate ion in the apatite structure of the 0.5Cu-OH sample, as reported above; the elimination of this ion is accompanied by a release of CO_2_ starting from 600 °C [22]. Reflections of the β-Ca_3_(PO_4_)_2_ phase in the 0.5Cu-OH sample were not detectable in the entire temperature range. This is because in this sample, copper ions are introduced at a greater-than-stoichiometric ratio (as an additive to the reagents used for reaction 1 in Table 1). Hence, there is no shortage of calcium cations in this sample (k = Ca/P = 1.67) after the exit of copper cations. At 1000 °C, the 0.5Cu-OH sample manifests an enlargement of the lattice parameters of apatite relative to those of unsubstituted HA (Figure 8a) as well as a decline in the CuO concentration (Table 3). These observations match the behavior of the 0.5Cu-Ca sample. Consequently, in 0.5Cu-OH, copper ions at high temperatures also return to the HA lattice and end up in the hydroxyl channel according to the mechanism described in Formula (6). 

The behavior of the coherent scattering region in the HA phase is almost identical among the studied samples (Figure 8b). When the samples are heated to 600 °C, the size of crystallites stays virtually unchanged. Next, there is intensive growth of crystallites up to a size of ~400 nm. The slight difference in crystallite size at 1200 °C can be explained by the presence of the β-Ca_3_(PO_4_)_2_ phase, which prevents the growth of Cu-HA crystallites in sample 0.5Cu-Ca.

Thus, from the data in Table 3, we can deduce that the synthesized Cu-HA samples, which were single-phase before heating, underwent phase transformations during the heating. In contrast to 0.5Cu-Ca, the 0.5Cu-OH sample returned to the single-phase state at 1100 °C.

### 3.3. Crystal Structure of the Cu-HA Samples after High-Temperature Treatment

**Figure 9:**
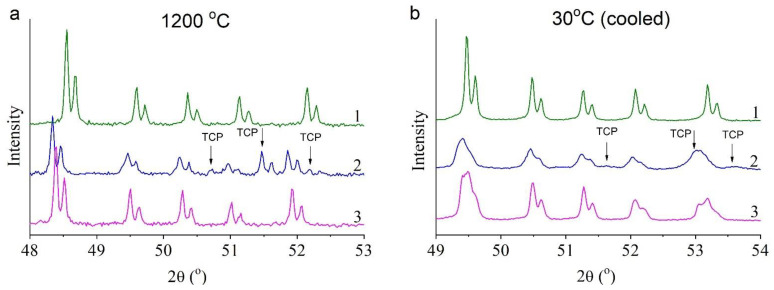
XRPD patterns of samples 0.0Cu (**1**), 0.5Cu-Ca (**2**), and 0.5Cu-OH (**3**) at 1200 °C (**a**) and after cooling to 30 °C (**b**).

**Figure 10:**
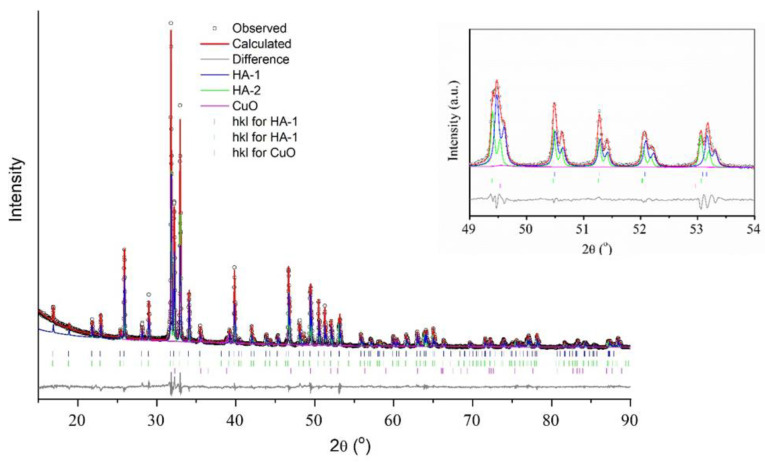
The Rietveld refinement plot for the 0.5Cu-OH sample.

In situ XRPD patterns indicated that in contrast to 0.5Cu-Ca, samples 0.0Cu and 0.5Cu-OH are single-phase at 1200 °C (Figure 9a). After cooling of the samples, one may observe that in 0.5Cu-Ca, there is a notable broadening of reflections of both the apatite phase and of tricalcium phosphate [β-Ca_3_(PO_4_)_2_; Figure 9b]. In the 0.5Cu-OH sample, which is single-phase at 1200 °C, splitting of some reflections of the HA phase is observed (Figure 9b). Rietveld refinement (Figure 10) shows that during the cooling of the 0.5Cu-OH sample, the Cu-HA phase decomposes into two phases having different lattice parameters (Table 4). The concentration of copper ions was determined based on the assumption that linear oxocuprate groups are formed in the hydroxyl channel [11]. The refinement results uncovered differences among the apatite phases in occupancy rates of copper ions (Table 4), in agreement with ref. [10]. The higher the copper content in the hydroxyl channel, the greater the lattice parameters of Cu-HA.

It is known that during the cooling of dehydroxylated HA in ambient air, the lost hydroxyl groups are restored, i.e., rehydroxylation takes place [23]. This is because reaction 7 is reversible; hence, higher water vapor pressure and a decrease in the temperature promote the incorporation of water into the crystal lattice. It is probable that the rehydroxylation, which proceeds most actively on the surface of particles, leads to the displacement of oxocuprate groups from surface layers of the particles, concurrently with the formation of nanosized CuO particles. Accordingly, reaction 8 is also reversible and depends on saturated vapor pressure. As a consequence of this process, a phase emerges having a lower copper content (probably the surface layer of the particle) and a higher copper content (the core of the particle); as a result, the coherent scattering region of substituted-HA particles diminishes considerably, while crystallite size decreases. Judging by the occupancy of copper ions, in the 0.5Cu-OH sample after heating to 1200 °C, the cooled sample contains the following phases of Cu-HA: Ca_10_(PO_4_)_6_(OH)_1.72_(CuO_2_)_0.14_ and Ca_10_(PO_4_)_6_(OH)_0.76_(CuO_2_)_0.62_.

A similar phenomenon of separation of Cu-substituted apatite into phases containing different copper concentrations, accompanied by a release of the CuO phase, has been documented by the authors of ref. [11], where the material was also slowly cooled in a high-temperature chamber. Ex situ heating with simultaneous air quenching enables researchers to obtain a single-phase material [12]. On the other hand, Karpov et al. have stated that the heating of a sample of certain composition to 1300 °C with subsequent slow cooling down to room temperature makes it possible to grow stand-alone crystals of Ca_10_(PO_4_)_6_Cu_0.27_O_0.86_H_y_ [13].

In the 0.5Cu-Ca sample, which contained the β-Ca_3_(PO_4_)_2_ phase at 1200 °C, the apatite phase did not split in two during cooling after the in situ experiment, likely owing to the low concentration of copper cations present in it (Table 4) because some of them are located in the β-Ca_3_(PO_4_)_2_ phase, as mentioned above. Upon cooling, copper oxide is released within this sample as well. The crystal lattice parameters of the β-Ca_3_(PO_4_)_2_ phase are less than those usually seen in unsubstituted β-Ca_3_(PO_4_)_2_ [27,28], thereby confirming the presence of copper cations in β-Ca_3_(PO_4_)_2_ at the positions of calcium cations, which possess a larger ion radius. During the cooling, less CuO separated out in the 0.5Cu-Ca sample than in the 0.5Cu-OH sample (Table 3). The calculated chemical formula of the Cu-HA phase in sample 0.5Cu-Ca in its cooled state is Ca_10_(PO_4_)_6_(OH)_1.32_(CuO_2_)_0.34_.

Colors of the samples changed substantially after the in situ experiments. Samples containing copper ions before the heating had a gray tint, whereas after heating to 1200 °C and subsequent cooling, they turned dark brown with a lilac tint. The brown color of the samples can probably be ascribed to the presence of copper (II) oxide, and the lilac hue is due to the oxocuprate groups in the Cu-HA lattice, as also demonstrated in ref. [29].

## 4. Conclusions

In the presented work, for the crystal lattice of HA, two types of substitution with copper ions were investigated. It was shown that with the help of soft mechanochemical synthesis, one can obtain Cu-HA with doping ions taking the place of calcium ions. After 30 min of mechanochemical processing of an appropriate mixture of initial reagents in a planetary ball mill, Cu-HA was successfully prepared with the degree of substitution (x) of calcium cations by copper cations at 0.5. Substitution of the hydroxyl group by this method of synthesis was unsuccessful.

For the first time, in situ diffraction analyses were performed on Cu-HA samples with (Ca + Cu)/P ratios of 1.75 and 1.67. It was established that at (Ca + Cu)/P = 1.75, the sample after the synthesis is single-phase Cu-HA, in which copper cations replace calcium cations. During heat treatment, starting from 600 °C, copper cations leave the HA structure with a release of a copper (II) oxide phase, while the crystal lattice of HA is preserved. The amount of the released CuO starts to diminish at 1000 °C, and CuO is undetectable at 1100 °C. Under these conditions, dehydroxylation of HA takes place, giving rise to OH group vacancies in the hydroxyl channel, thereby driving a reverse process: CuO diffusion into the HA lattice. Oxocuprate groups (O–Cu–O)^2−^ are formed in the hydroxyl channel. The resultant substance is cuprate-substituted HA with the general chemical formula Ca_10_(PO_4_)_6_(OH)_2−2x_(CuO_2_)_x_. After the cooling of this material, the CuO phase appears, indicative of the reverse process of diffusion of copper oxide in conjunction with the rehydroxylation of apatite. Nonetheless, some copper cations remain in the HA lattice. The cooled sample is a mixture containing 58 wt% Ca_10_(PO_4_)_6_(OH)_1.72_(CuO_2_)_0.14_, 41 wt% Ca_10_(PO_4_)_6_(OH)_0.76_(CuO_2_)_0.62_, and 1 wt% CuO.

At (Ca + Cu)/P = 1.67, the product of the mechanochemical synthesis is single-phase Cu-HA, where copper cations substitute for calcium cations. When this material is heated, starting from 700 °C, copper cations leave the HA lattice with a release of the CuO phase. Starting from 800 °C, a third phase begins: Cu-substituted tricalcium phosphate β-Ca_3_(PO_4_)_2_. With a further increase in temperature, the concentration of β-Ca_3_(PO_4_)_2_ increases, while the CuO concentration declines because reverse diffusion of CuO into the HA lattice occurs, with the formation of oxocuprate groups (O–Cu–O)^2−^ in the hydroxyl channel of HA and the emergence of a mixed phosphate. The cooled sample is a three-phase system containing 70 wt% Ca_10_(PO_4_)_6_(OH)_1.32_(CuO_2_)_0.34_, 29 wt% Cu-substituted β-Ca_3_(PO_4_)_2_, and 1 wt% CuO.

Thus, by in situ diffraction analyses of cuprate-substituted HA, it is shown for the first time that the highest concentration of copper cations in the HA lattice is attained at a high temperature. Slow cooling of the material is accompanied by rehydroxylation, which promotes the exit of some proportion of the dopant in the oxide form.

## Figures and Tables

**Table 1:** Expected reactions of mechanochemical synthesis.

Reaction ID	Reaction	K		Sample Name
		Ca/P	(Ca + Cu)/P	
1	6CaHPO_4_ + 4CaO → Ca_10_(PO_4_)_6_(OH)_2_ + 2H_2_O	1.67	1.67	0.0Cu
2	6CaHPO_4_ + 3.5CaO + 0.5CuO → Ca_9.5_Cu_0.5_(PO_4_)_6_(OH)_2_ + 2H_2_O	1.58	1.67	0.5Cu-Ca
3	6CaHPO_4_ + 4CaO + 0.5CuO → Ca_10_(PO_4_)_6_(OH)_1_Cu_0.5_O + 2.5H_2_O	1.67	1.75	0.5Cu-OH

**Table 2:** Structural characteristics of the synthesized samples.

Sample Name	*a* (Å)	*c* (Å)	Crystallite Size (nm)
0.0Cu	9.437 (1)	6.892 (1)	24.9 (2)
0.5Cu-Ca	9.435 (1)	6.881 (1)	20.2 (2)
0.5Cu-OH	9.432 (2)	6.887 (1)	20.2 (2)

Note: The estimated standard deviations of the refined values are in parentheses.

**Table 3:** Concentrations (C) of impurity phases and changes in crystallite size (CS) of the substituted-HA samples during the in situ experiment.

Temp. (°C)	Impurity Phases in the Samples							
	0.5Cu-Ca (Ca + Cu)/P = 1.67				0.5Cu-OH (Ca + Cu)/P = 1.75			
	β-Ca_3_(PO_4_)_2_		CuO		β-Ca_3_(PO_4_)_2_		CuO	
	C (wt%)	CS (nm)	C (wt%)	CS (nm)	C (wt%)	CS (nm)	C (wt%)	CS (nm)
500	–	–	–	–	–	–	–	–
600	–	–	–	–	–	–	2.8 (2)	15.8 (5)
700	–	–	1.1 (3)	52 (17)	–	–	3.5 (3)	19.2 (4)
800	17.0 (8)	134 (9)	2.4 (4)	45 (8)	–	–	3.5 (2)	32.1 (4)
900	25.6 (8)	220 (26)	2.0 (3)	105 (22)	–	–	3.5 (2)	32.0 (4)
1000	27.0 (8)	288 (22)	0.7 (4)	77 (49)	–	–	2.9 (2)	56.2 (8)
1100	30.9 (5)	444 (43)	–	–	–	–	–	–
1200	33.7 (5)	593 (66)	–	–	–	–	–	–
500	–	–	–	–	–	–	–	–

Note: The estimated standard deviations of the refined values are in parentheses.

**Table 4:** Concentrations of impurity phases and changes in the coherent scattering region within the cooled samples in the in situ experiment.

		Sample Name		
		0.0Cu	0.5Cu-Ca	0.5Cu-OH
HA-1/HA-2 *	C (wt%)	100	70.2 (6)	58.2 (4)/40.8 (4)
	*a* (Å)	9.4224 (1)	9.4274 (3)	9.4281 (6)/9.4318 (4)
	*c* (Å)	6.8843 (1)	6.9006 (3)	6.8922 (5)/6.9058 (4)
	Crystallite size (nm)	384 (25)	139 (3)	199 (8)/362 (28)
	Cu occupancy	–	0.34 (2)	0.14 (2)/0.62 (4)
	Calculated chemical formula	Ca_10_(PO_4_)_6_(OH)_2_	Ca_10_(PO_4_)_6_(OH)_1.32_Cu_0.34_O_0.68_	Ca_10_(PO_4_)_6_(OH)_1.72_Cu_0.14_O_0.28_/Ca_10_(PO_4_)_6_(OH)_0.76_Cu_0.62_O_1.24_
**β-Ca_3_(PO_4_)_2_**	C (wt%)	–	29.2 (6)	–
	*a* (Å)	–	10.4050 (7)	–
	*c* (Å)	–	37.426 (3)	–
	Crystallite size (nm)	–	127 (7)	–
**CuO**	C (wt%)	–	0.6 (3)	1.0 (4)
	Crystallite size (nm)	–	170 (65)	58 (16)
**Reliability factor R_wp_**		5.2	5.8	4.8

* If two HA phases are present in a sample, then parameters of the second HA phase are given after a forward slash.

## Data Availability

The raw/processed data required to reproduce these results are included in the Section 2.
